# Reasons for living and hope as the protective factors against suicidality in Chinese patients with depression: a cross sectional study

**DOI:** 10.1186/s12888-016-0960-0

**Published:** 2016-07-20

**Authors:** Xingwei Luo, Qin Wang, Xiang Wang, Taisheng Cai

**Affiliations:** Medical Psychology Center, The Second Xiangya Hospital, Central South University, Changsha, Hunan 410011 China; Medical Psychology Institute of Central South University, Changsha, Hunan 410011 China; The Third Experimental Primary School, Rizhao, Shandong 276800 China; Medical Institute of Psychology, Secong Xiangya Hospital of Central South University, 139 Renmin Road, Furong District, Changsha, 410011 China

**Keywords:** Depression, Reasons for living, Hope, Suicidal ideation, Suicide attempt

## Abstract

**Background:**

The risk factors of suicidal ideation and attempts have been discussed in many researches. Few studies have examined reasons for living and hope as protective factors against suicide in a clinical population. It is unclear if these factors help to reduce suicide rates in patients with depression. The study aimed to assess the role of reasons for living and hope in the identification and reduction of suicidality and explore the influence of reasons for living or hope in the transition from suicidal ideation to suicide attempts.

**Methods:**

Patients with depression (*N* = 115) completed the Beck Depression Inventory, Reasons for Living Inventory, and Adult Suicidal Ideation Questionnaire.

**Results:**

There were significant correlations among depression, hope, total reasons for living, and suicidal ideation and attempts. Further, after controlling for depression, reasons for living and hope had significant main effects on suicidal ideation. Hope also had a significant predictive effect in the transition of suicidal ideation to suicide attempt.

**Conclusions:**

We concluded that reasons for living and hope may protect against suicidal ideation and attempts in patients with depression. Especially hope could reduce the possibility of suicide attempt.

## Background

Suicide is a serious public health problem around the world. The overall suicide rate in China was between 17 and 23 people per 100,000 more than 20 years ago, with about 287,000 people dying due to suicide every year. The number of deaths attributable to suicide has decreased in recent years, but it is still the fifth leading cause of death overall and a primary reason for death among people aged between 15 to 34 years in China [1]. Researchers have found that among those who die by suicide, nearly 90 % were previously diagnosed with a mental disorder [2–4], and that depression is the most common among these disorders [5]. Several recent national and regional epidemiologic studies of mental disorders have revealed that the lifetime prevalence of depression in China ranges from 1.2 to 8.6 % [6].

Many studies have shown that depression has a close relationship with suicidal ideation [7–9], with the latter being defined as the first step of suicidal behavior [10] and a risk factor for later suicide attempts [11, 12]. Britton et al. [7] found that both hopelessness and depression might lead to suicidal ideation, and other researchers stated that additional risk factors were neuroticism, negative life events, and a perceived lack of social belonging [13, 14]. Among these, the depression is the most important factor in predicting an increased likelihood of suicidal ideation [8, 15]. Furthermore, the severity of depression and suicidal ideation could be used as predictors of a future suicide attempt [16]. Therefore, it is necessary to find out the relationship among them.

Most researchers have focused on the risk factors of suicidal ideation and attempts, instead of on positive emotions that reduce these risks. With the rise of positive psychology over the last two decades, more emphasis has been placed on the influence of positive attitudes, such as reasons for living and hope, on suicidal ideation. There is some evidence that lower suicidal ideation is related to positive factors, such as feeling satisfied with interpersonal relationships, feeling useful to family and friends, feeling that life has meaning, and pursuing a meaningful life [17, 18]. Moreover, Linehan et al. [19] found those who attempted suicide were lacking in adaptive and survival faith, while these types of faith were common among the general population. Examples of these beliefs are the perceptions that no matter how bad life is, there are still things to enjoy, and that one’s circumstances will get better in the future. These life goals and expectations are termed reasons for living, which is a concept that researchers have examined in many different populations. Their results show that the reasons for living of survival and coping beliefs, and responsibility to family are associated with lower suicidal ideation [7, 20–23]. Individuals who have experienced suicidal ideation and engaged in suicide attempts have been found to have lower moral objections [24]. Fear of social disapproval has not been linked with suicidal ideation [20], but a relationship was found with actual suicide attempts [25]. Child-related concerns are more likely to influence older, rather than younger people [26]. However, there is a divergence in results with regard to the relationship between fear of suicide and suicidal ideation [7, 20, 21].

Another positive factor is the hope that has been acknowledged for a very long time. Hope is a future-oriented attitude that helps individuals to get through times of crisis by projecting thoughts of a future that is full of possibilities. Hanna [27] regarded the idea of hope as a marker of lower suicidal ideation, stating that a person who is hopeful is a person who wishes to live, and other studies have supported this idea [28, 29]. Researchers have also found that hope predicts lower levels of perceived burdensomeness and thwarted belongingness, which have been posited to create the desire to die by suicide [30], but higher levels of acquired capability to enact lethal self-injury [28, 31]. Recent research has found that hope can act as a resilience factor that buffers the impact of hopelessness on suicidal ideation [32].

Although existing studies about reasons for living and hope have provided promising results and the differences between suicidal ideation and attempts have been examined, there is still a need for additional research for several reasons. First, few studies have examined reasons for living and hope as protective factors against suicide in a clinical population. It is especially unclear if these factors help to reduce suicide rates in patients with depression. Further, most of the current literature about potential risk compares suicide attempters and suicide ideators to nonsuicidal individuals, but not to each other. Thus, they lacks specificity in distinguishing which variables are associated with suicidal ideation and which are associated with suicide attempts [33]. As far as we know, no studies have explored the influence of reasons for living or hope in the transition from suicidal ideation to suicide attempts. In order to separate the factors for suicidal thinking and for suicidal action, Klonsky and May [34, 35] suggest that an “ideation-to-action” framework should guide all suicide research. All explanations for suicide need to be clear as to whether they address the risk for (a) suicidal ideation, (b) suicide attempts in those ideating, or (c) both. In such a framework, the following hypotheses were formed with regard to patients with depression: (1) Reasons for living and hope will be negatively associated with depression, suicidal ideation, and suicide attempts, (2) Reasons for living and hope and their subscales will negatively predict suicidal ideation, (3) Reasons for living and hope will prevent the transition from suicidal ideation to suicide attempts.

## Methods

### Participants

Participants were 115 outpatients with depression who were undergoing treatment at a psychological clinic in Xiangya Second Hospital at Central South University in China. The mean age of the sample was 27.0 years (±5.4, range = 22 to 59 years), and 40.0 % (*n* = 46) were male, with the remaining 60.0 % (*n* = 69) being female. Seventy four percent were single and 25 % were married. Fifty seven percent completed junior high education, 26 % completed high school education and 16 % completed undergraduate education. The mean total household income was about the equivalent of middle-income levels in China. The current depressive symptom severity was in the moderate-to-severe range as measured by the BDI(25.5 ± 10.1). Fifty seven patients(49.6 %) had suicidal ideation and 25 patients (22 %) reported a previous suicide attempt. The mean duration of depression was about 1 year. In terms of treatment, 68 % of cases were the first time to receive treatment and others were on at least one antidepressant (SSRI/SNRI).

All patients met the Diagnostic and Statistical Manual of Mental Disorders (4th ed.) criteria for a current major depressive episode and Beck Depression Inventory [36] scores ≥ 21. Exclusion criteria included current substance or alcohol abuse, diagnosis with a neurological illness, serious physical disease, and a history of craniocerebral trauma, mental retardation, other organic diseases, drug-induced depression, and drug-induced bipolar disorder.

### Research procedure

The G*Power 3.1 software was used to estimate the study sample size according to the research design. After being informed of the research purposes and giving their permission, participants were asked to sign the informed consent statements approved by the university Institutional Review Board. Then a comprehensive clinical interview based on DSM-IV diagnosis of depression was done together with self-report questionnaires detailed below. The interviews were conducted by an experienced psychiatrist during the course of the study. Lifetime suicide attempt was assessed through the interview and recorded with Yes/No. Every assessment was done not more than 24 h. Specially, our paper reporting adheres to the STROBE guidelines of observational studies.

### Instruments

#### Beck depression inventory (BDI, [36])

The BDI includes 21 symptom checklist items that represent the severity of symptoms, each of which is scored on a scale from 0 = low to 3 = high. Total scores range from 0 to 63, with a higher score indicating more severe depressive symptoms, and ranges are divided as follows: ≤ 4 = very minor depression or no depression; 5–13 = mild depression; 14–20 = moderate depression; ≥ 21 = severe depression. In this study, the internal consistency coefficient of the BDI was 0.86.

#### Snyder hope scale (SHS, [37])

The SHS is a 12-item self-report measure that assesses agency (four items) and pathways (four items) components of hope, and also contains four filler items. Respondents rate each item in terms of the degree to which it is true or not true of them, using a 4-point Likert scale (0 = definitely false, 3 = definitely true). Items from the agency and pathways subscales are summed to yield a total hope score that appears to be reasonably temporally stable, with test-retest correlations ranging from 0.85 over a 3-week period to 0.82 over a 10-week period [37].

#### Reasons for living inventory (RFL)

The RFL was compiled by Linehan and colleagues in 1983, and the Chinese version was developed by Deng, Xiong, and Lin [38]. In this study, we used the Chinese version, which includes 48 items divided across six dimensions, that is, survival and coping beliefs (SCB, 24 items), responsibilities to family (RF, 7 items), moral objections (MO, 4 items), fear of suicide (FS, 7 items), fear of social disapproval (FSD, 3 items), and child-related concerns (CRC, 3 items). Responses are made using a 6-point Likert scale that ranges from 1 = not important (not a reason for me to avoid suicide) to 6 = extremely important (a significant reason not to commit suicide). Participants were asked to choose the response that best fit their actual situation. Higher scores mean that the individual has more reasons for living. The internal consistency coefficient of the scale’s Chinese version was found to be 0.94, and in this study, it was 0.83.

#### Adult suicidal ideation questionnaire (ASIQ, [39])

The ASIQ consists of 25 items that are rated on a 7-point scale, ranging from 0 = have never thought about this to 6 = think about this almost every day, and a higher score indicates stronger suicidal ideation. In adults, the demarcation of suicidal ideation is set at 23 points [39], such that a score higher than or equal to 23 indicates the presence of suicidal ideation, while a score under 23 indicates no suicidal ideation. In this study, the scale’s internal consistency coefficient was 0.90.

### Data analysis

The collected data were analyzed using the Statistical Package for the Social Science 18.0(SPSS 18.0). Pearson’s correlation coefficient was used in analyzing the relationships among depression, hope, reasons for living and suicidal ideation. Further, hierarchical regression analysis was used to explore the impact of hope and reasons for living on suicidal ideation after controlling for depression. Finally, logistic regression analysis was used to explore the influence of the factors of depression, hope, and reasons for living on suicide attempts. The level of statistical significance was set at 5 %.

## Results

### Descriptives and intercorrelations

Demographic variables were not significantly related to suicidal outcomes. A descriptive analysis of all variables and the bivariate correlations among the main variables of interest are shown in Table 1. The results showed that there were significant correlations among depression, hope, agency, pathways, total reasons for living, SCB, RF, MO, CRC, and suicidal ideation (*r*s = −0.201 to −0.534). There were also significant correlations among, hope, agency, pathways, total reasons for living, survival and coping beliefs (SCB), responsibility to family (RF), child-related concerns (CRC) and depression (*r*s = −0.195 to −0.443).

**Table 1:** Descriptives and correlations among depression, reasons for living, hope and suicidal ideation

			HOPE			RFL						
Variable	SI	BDI	Total	Agency	Pathways	Total	SCB	RF	MO	FS	FSD	CRC
Frequency (%)/mean	32.91	25.49	33.29	16.33	16.96	188.13	96.47	30.57	10.47	26.49	12.42	11.44
SD	34.58	6.11	11.69	6.45	6.67	46.80	29.06	7.69	5.16	7.44	4.19	7.75
SI	1											
BDI	0.470^**^	1										
HOPE-TOTAL	−0.349**	−0.393**	1									
Agency	−0.391**	−0.443**	0.887**	1								
Pathways	−0.233*	−0.269**	0.895**	0.588**	1							
RFL-TOTAL	−0.449^**^	−0.271^**^	0.163	0.234*	0.060	1						
SCB	−0.534^**^	−0.352^**^	0.188*	0.276**	0.064	0.948^**^	1					
RF	−0.201^*^	−0.198^*^	0.228*	0.263**	0.145	0.698^**^	0.557^**^	1				
MO	−0.263^**^	−0.007	0.012	0.084	−0.061	0.701^**^	0.558^**^	0.457^**^	1			
FS	−0.079	0.031	−0.075	−0.057	−0.077	0.637^**^	0.488^**^	0.298^**^	0.536^**^	1		
FSD	−0.137	−0.057	−0.01 2	0.020	−0.041	0.634^**^	0.526^**^	0.496^**^	0.415^**^	0.393^**^	1	
CRC	−0.307^**^	−0.195^*^	0.207*	0.179	0.190*	0.615^*^*	0.502^**^	0.456^**^	0.470^**^	0.319^**^	0.286^**^	1

*SI* suicidal ideation, *BDI* Beck depression inventory, *HOPE* Snyder hope scale, *RFL* reasons for living, *SCB* survival and Coping beliefs, *RF* responsibility to family, *MO* moral objections, *FS* fear of suicide, *FSD* fear of social disapproval, *CRC* child-related concerns

* *p <* 0.05, ** *p <* 0.01

### Hierarchical regression analysis of factors affecting suicidal ideation

Hierarchical regression analysis was used to explore the influence of hope and reasons for living on suicidal ideation. In the first model, the depression was a strong predictor of suicidal ideation as expected(β = 0.47, *R*^2^ = 0.220, *p* < 0.01). In the second model, hope, SCB, RF, CRC, MO were entered as independent variables. The RF, CRC and MO were not significant, but hope and SCB remained a negative effect on suicidal ideation after the depression was controlled. These results are shown in Table 2.

**Table 2:** Hierarchical regression analysis of suicidal ideation

Variables	B	β	t	R^2^	△R^2^	F
Step 1				0.220	0.220	28.69^**^
BDI	1.42	0.47	5.36^**^			
Step 2				0.374	0.154	9.66^**^
HOPE	−0.71	−0.27	−2.99^**^			
SCB	−0.36	−0.33	−2.84^**^			
RF	0.64	0.16	1.56			
MO	0.05	0.01	0.07			
CRC	−0.68	−0.11	−1.03			

### Factors influencing suicide attempts

We found that 25 (22 %) of the patients in our survey had attempted suicide. A further 57 patients with suicidal ideation were divided into groups who had and had not attempted suicide. Logistic regression was applied, with suicide attempts as the dependent variable and depression, four RFL subscales, hope as the independent variables. The results revealed that only hope remained significantly associated with suicide attempts (odds ratio = 0.94, 95 % CI [0.88–0.99], *p* = 0.032; see Table 3).

**Table 3:** Logistic regression analysis with suicide attempts as the dependent variable

	OR	95 % CI	Wald	*p*
BDI	1.02	[0.94–1.10]	0.21	0.647
SCB	0.99	[0.96–1.02]	0.39	0.533
RF	1.02	[0.93–1.11]	0.11	0.746
MO	1.01	[0.86–1.20]	0.03	0.870
CRC	0.97	[0.82–1.15]	0.13	0.716
HOPE	0.94	[0.88–0.99]	4.61	0.032*

## Discussion

The study results show that reasons for living, SCB, RF, CRC, MO, and hope, agency, pathways were negatively associated with suicidal ideation, while fear of suicide and fear of social disapproval did not influence suicidal ideation. We also found that SCB and hope had significant negative effects on suicidal ideation. The logistic regression analysis showed that hope was a protective factor, preventing suicidal ideation from translating into a suicide attempt in our patients with depression.

Reasons for living had negative effect on suicidal ideation, indicating that reasons for living may serve as a protective factor to reduce suicidal ideation. This is consistent with the findings in previous studies [7, 20, 22, 23, 40]. As reasons for living are considered to be related to life goals and expectations, they act as a protective factor against suicidal ideation. Patients with depression often have many negative and pessimistic thoughts, feel life has no meaning or value, lack confidence in the future, and, therefore, experience suicidal ideation. Those patients with a greater number of reasons for living were usually more inclined to acknowledge the existence of these reasons. Even if the depression made them feel pain and helplessness, their ability to endure pain was significantly greater than that in patients with fewer reasons for living, and this evoked a positive willingness and motivation to change their situation. The emphasis on reasons for living coincided exactly with the logotherapy of Viktor Frankl [41], who believed that “life can be made meaningful by the attitude we choose toward suffering,” (p. 37), such as the exploration and pursuit of the meaning of and value placed on life meeting people’s basic spiritual needs. Individuals with fewer reasons for living need help to discover a meaning for their life and to ensure that they feel hope about the future, thus reducing the risk of suicidal ideation.

Our study also found that the SCB dimension of reasons for living was closely and negatively associated with predicting suicidal ideation, which supports the findings of previous studies [7, 21]. The consistency of these findings indicates that SCB is important for survival because it helps individual confront stress and psychological crises, and provides the confidence to deal with issues and form positive expectations for the future. The former is similar to the concept of efficacy, while the latter relates to a sense of hope [19]. High levels of efficacy can predict good mental health [42] and reduce psychological problems [43]. Similarly, a sense of hope is a protective factor against suicide [44]. As a type of positive attitude towards life, sense of hope could help people through a period in their life that is full of frustration because they will imagine a future full of possibilities and be unlikely to take an extreme action, like suicide, to solve their problems. Therefore, enhancing SCB is an effective way to reduce the risk of suicidal ideation.

Further, higher RF and CRC, which measure the importance one places on their family members, were associated with lower suicidal ideation, in line with the findings of previous researchers [20, 22]. On the one hand, a strong sense of responsibility for one’s child and family would influence reflections on one’s own situation [45]; on the other hand, patients with a more intimate relationship with family members have been found to have lower suicidal ideation [46]. Further, when they have a longer term and stronger attachment to their children, they are not likely to end their lives because children provide a compelling obligation to continue living [26]. For patients with depression, even those with serious depression, a sense of responsibility for caring for their children and maintaining their reputation means that they will not die by suicide. In most cultures, there is a strong focus on family values; people pay a lot of attention to family responsibility and prefer to put personal emotional issues aside. The responsibility for taking care of one’s family is, thus, stronger than suicidal ideation, thereby protecting the individual against this factor. Similarly, higher MO led to lower suicidal ideation, which supports the findings of some previous studies [24, 47], proved that objections to suicide on moral grounds may also serve as a protective factor against suicidal behavior in depressed inpatients.

Of the six dimensions of reasons for living, fear of suicide and fear of social disapproval were not found to have a correlation with suicidal ideation, a finding that is consistent with those of most previous studies [20, 21]. We predicted that that fear of suicide and fear of social disproval would be related to concerns about the suicide act and its consequences. As forms of negative emotion, they did not reduce suicidal ideation but did reduce the likelihood of an individual to die by suicide [25]. Britton et al. [7] found that in an elderly population, fear of suicide was negatively correlated with suicidal ideation. This differs from the general adult population and groups of younger people, perhaps because older people have a more intense fear of suicide and death and, therefore, try to avoid thoughts related to suicide.

A higher severity of suicidal ideation, based on scores on the ASIQ, was significantly associated with lower hope in the correlation analysis and single predictive models. This result supports Snyder’s [48] (p. 267) hope theory, in which it is stated that when people have met “profound, chronic and seemingly unending goal blockages…may abandon their usual life goals in favor of a suicide goal” (p. 267). It is generally known that learned helplessness is very common in patients with depression, who typically experience long-term goal blockages and do not feel hope. In this case, they would consider suicide as the final goal, leading to the generation of suicidal ideation. Therefore, we consider hope to be the protective factor against suicidal ideation. Further, our results showed that of the two dimensions of hope, agency played a more important role in the course of suicidal ideation than pathways did. Snyder [49] stated that hope theory is anchored by a goal; that is, when we are motivated to accomplish something, we are often confident in ourselves and full of expectations about the future [44]. In this case, suicidal thoughts are rarely aroused. In contrast, pathways thinking relates to the ability to achieve goals and so the correlation with suicidal ideation is relatively weaker.

To our knowledge, this is the first study of the role of hope in the process of transition from suicidal ideation to suicide attempts. As we predicted, hope is a protective factor against suicide attempts in patients with depression. This differs from previous research in which it was found that hope was a significant and positive predictor of the capability to complete suicide [28, 31, 50]. This may be partly because of the difference in research participants, whereby in our study, the patients had suffered from continuous depressive symptoms. In line with this, Snyder [37, 48] theorized that people act on their suicidal intent to escape from unbearable pain, so when they feel hopeful about other things they will put their energy toward these goals to relieve the pain. In contrast, for the general population, these goals may become a burden and expose them to pain. This tells us that if a person has other options, suicide will not be the preferred goal, and hope provides the possibility of a variety of options. Besides, a recent meta-analysis [33] has indicated that hopelessness was slightly higher among suicide attempters compared to suicide ideators. It proves the influence of hope on suicide attempts from a very opposite aspect. Therefore, hope can be an effective method of suicidal behavior prevention.

Although this article provides empirical evidence that reasons for living and hope can be used to understand suicidal ideation and suicide attempts in patients with depression, there are limitations to this study that deserve comment, and we also have suggestions for areas for future study. First, our study included a relatively small sample of patients with depression and it is unknown whether the results can be generalized to other such patients or people without depression. Second, given our cross-sectional design, the temporal ordering of constructs cannot be determined. Prospective event-based assessments are needed to clarify the role of reasons for living and hope in the transition from suicidal ideation to suicide attempt. Finally, we used clinical interviews when evaluating suicide attempts, which may have mean that some valuable information was missed. Future studies should use validated structural interview measures to assess suicide attempts.

## Conclusions

This study still enriched the theory on reasons for living and hope, further clarifying the relationship between these factors and suicidal ideation and attempts. In particular, we found that hope plays an important role in the transition from suicidal ideation to actual attempts in patients with depression. We suggest that future studies could test changes in brain function from the perspective of cognitive neuropsychology. In terms of clinical implications, when suicide appears to be the only possible solution for patients with depression, it is important to help him or her to find meaningful things in life, make future plans, and identify alternative solutions, thus protecting high-risk groups from suicide. Because suicidal action is preceded by goal setting, pathways thinking, and agency, there are possibilities for intervention in each of these areas [37].

## Abbreviations

ASIQ, adult suicidal ideation questionnaire; BDI, Beck depression inventory; CRC, child-related concerns; FS, fear of suicide; FSD, fear of social disapproval; MO, moral objections; RF, responsibility to family; RFL, reasons for living inventory; SA, suicide attempt; SCB, survival and coping beliefs; SHS, Snyder hope scale; SI, suicidal ideation
